# Digital infrastructure policies, local fiscal and financing constraints of Non-SOEs: Evidence from China

**DOI:** 10.1371/journal.pone.0327294

**Published:** 2025-07-30

**Authors:** Yuwei Liu, Sheng Ma, Mei Sun

**Affiliations:** Chengdu University, Chengdu, China; Southwestern University of Finance and Economics, CHINA

## Abstract

Digital infrastructure serves as a cornerstone of urban digital transformation and smart city development, yet its implications for local fiscal systems and micro-level enterprises remain underexplored. This study empirically investigates the impact of digital infrastructure policies on the financing constraints of Non-SOEs in China. The contributions of this paper are as follows: (1) *Theoretical innovation*: It develops a comprehensive theoretical framework connecting macro-level digital policies, regional fiscal dynamics, and micro-enterprise financing constraints, offering a novel perspective on how macro policies influence micro-enterprises. (2) *Systemic analysis*: It enhances understanding of the systemic effects of digital infrastructure policies by demonstrating their ability to alleviate financing constraints through mitigating fiscal burdens, improving budgetary revenue quality, and strengthening regional financial development. Analysis of heterogeneity reveals that digital infrastructure policies implemented by Chinese provincial governments are particularly effective. (3) *Practical insights*: It offers practical guidance for policymakers to design targeted strategies that reduce financing constraints and support private sector growth in the digital economy. Non-SOEs in the growth and decline periods benefit more from digital infrastructure due to higher financing demands. Non-SOEs independent of SOEs in the supply chain are more responsive to digital infrastructure, effectively alleviating financing constraints. Moreover, the construction of digital infrastructure is highly conducive to the attraction of Non-SOEs and does not result in the vertical imbalances in local fiscals that are associated with traditional infrastructure construction. This evidence offers valuable guidance for local governments to optimize digital transformation policies and foster private economy growth.

## 1. Introduction

Digital transformation serves as a critical foundation for advancing urban development through four interconnected dimensions: digital industrialization, industrial digitization, digital governance enhancement, and digital value creation. This technological paradigm shift plays a pivotal role in establishing China’s modern economic framework while particularly strengthening the competitive capabilities of Non-SOEs. All 31 provinces and cities in China have comprehensively launched digital transformation initiatives, with continuous optimization and phased implementation of digital infrastructure policies progressively broadening application scenario matrices. Through deepening technological integration, digital solutions are being extensively embedded within industrial operational ecosystems. This trend drives sustained expansion of digital transformation applications across urban industrial sectors.

The implementation of digital infrastructure policies imposes dual pressures on local fiscal. On the one hand, digital infrastructure policies strain local budgets. Substantial upfront capital requirements for hardware, communications systems, and network infrastructure (Wang et al., 2023) [1] risk exacerbating fiscal deficits in financially constrained regions, potentially distorting micro-enterprise operations through increased debt burdens. On the other hand, contrary to traditional infrastructure’s marginal user scalability with diminishing returns (Hussain and Jun, 2024; Pei et al., 2018; Luo and Fan, 2012) [2–4], digital infrastructure has infinite marginal benefit and no difficulty with diminishing marginal value: once operational, its outputs can be replicated at near-zero marginal costs despite substantial fixed investments. This economic characteristic enables digital infrastructure outputs to demonstrate diminishing marginal cost attributes (Pei et al., 2018) [3], ultimately benefiting both municipal local fiscal sustainability and enterprise viability. How would digital infrastructure policies impact local fiscal and therefore influence private enterprise? Addressing this issue could offer local governments insights into effectively managing their finances while investing in digital infrastructure. Additionally, it could offer valuable empirical evidence to encourage private enterprise financing. This study analyzes a sample of private listed companies in China’s A-share market from 2015 to 2021 to examine the impact of digital infrastructure policies on financing dynamics. Specifically, through manual content analysis of 31 provincial policy documents, we establish a significant negative correlation (β = −0.003, p < 0.01) between policy intensity and financing constraint severity. Mechanism analyses identify that digital infrastructure policies alleviate local fiscal burdens, improve the quality of fiscal revenue, and enhance the regional financial ecosystem, thereby easing financing constraints. Heterogeneity tests reveal that provincial-level policies exhibit 58.3% significantly stronger effects than other institutions. Additionally, growth/decline-stage firms show higher sensitivity to policy impacts. Furthermore, SOE-independent Non-SOEs demonstrate greater responsiveness. Notably, this paper confirms confirm digital infrastructure’s agglomeration effects, attracting enterprise relocation while dispelling fiscal imbalance concerns. Robustness checks employing instrumental variable approaches, Heckman two-step models, and variable substitution tests validate result consistency.

This study makes three principal contributions to the field: First, it advances theoretical understanding of digital infrastructure policy impacts by establishing an integrated framework that incorporates local fiscal dynamics, enterprise financing constraints, and policy implementation. Departing from conventional analyses of direct policy effects, our approach reveals how fiscal adjustments triggered by digital infrastructure policies reshape Non-SOEs’ credit accessibility and financing needs. This mechanistic perspective elucidates the transmission pathways through which macroeconomic policies influence microeconomic entities.

Second, it broadens the understanding of digital infrastructure policies. Since 2014, when the Central Committee of the Communist Party of China (CPC) comprehensively deployed the overall demands of digital infrastructure construction, local governments have gradually introduced various targeted policy campaigns; however, prior studies predominantly focus on isolated policy instruments (e.g., “Broadband China” initiatives) while neglecting the systemic nature of contemporary digital infrastructure programs that integrate planning, technological integration, and value-chain coordination (Cao et al., 2025; Gu et al., 2024; Jin et al., 2023) [5–7]. As the digital economy grows, digital infrastructure policies evolve into systematic projects that integrate planning, science, systematic approaches, and value. Consequently, research that employs granular policy text analysis of 31 provincial documents provides valuable insights for capturing the evolving systematicity of digital infrastructure policies, contrasting with earlier single-policy evaluations.

Third, the findings offer empirically grounded solutions for alleviating Non-SOEs’ financing constraints through macro-level institutional arrangements. The crucial mediating role of local fiscal architectures in transmitting macro-level digital policies to micro-enterprise financing outcomes remains underexplored, particularly regarding how fiscal burden redistribution and credit space reconfiguration modulate policy effectiveness. Our evidence demonstrates how digital infrastructure investments fundamentally restructure urban fiscal architectures, thereby creating sustainable financing channels. These insights enable policymakers to design multidimensional support systems that align digital transformation objectives with enterprise development needs across organizational lifecycles.

## 2. Literature review and research hypotheses

### 2.1. Negative exacerbation effects of digital infrastructure policies on Non-SOEs financing constraints

China’s 1994 fiscal decentralization reform established a structural disparity between local fiscal capacities and administrative authorities, creating structural *fiscal gaps* that trigger local vertical fiscal imbalance (Cai and Chen, 2023; Gu and Zhu, 2023) [8,9]. This institutional configuration compels local governments to pursue extra-budgetary revenue channels (Jiang, 2006) [10], while GDP-centric political promotion mechanisms further impact local fiscal conduct.

First, budgetary expenditure distortions emerge under dual fiscal-political pressures. local governments prioritize capital-intensive infrastructure investments as measurable economic growth indicators (Zhang et al., 2007) [11], initiating a self-reinforcing cycle of *competitive fiscal gaps* (Hong et al., 2018) [12]. Persistent infrastructure investments deplete budgetary allocations while expanding expenditure commitments, directly influencing local debt accumulation patterns (Meloni, 2016) [13].

Second, local financing vehicles (LFVs) facilitate massive debt issuance to meet public investment demands, concurrently crowding out private-sector credit availability and elevating financial market pressures (Cai and Chen, 2023; Yu and Kang, 2020) [14,15]. This credit displacement effect disproportionately impacts Non-SOEs within respective jurisdictions, triggering constrained access to financing.

**Fig 1:**
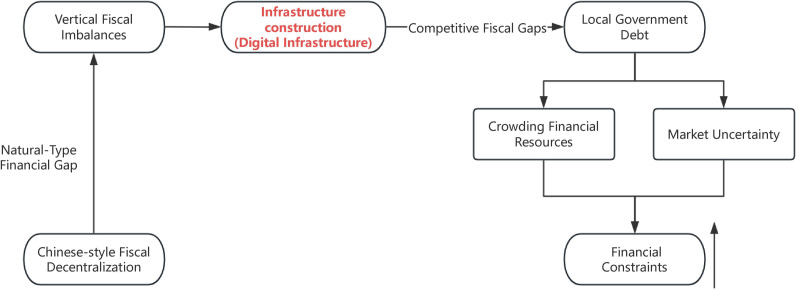
The Negative exacerbation effects of digital infrastructure policies on Non-SOEs financing constraints.

Third, vertical fiscal imbalances amplify regional economic uncertainties. Policy volatility stemming from debt-driven growth models induces investor risk aversion, particularly constraining Non-SOEs ‘ access to capital markets. The diagram illustrating the negative effects is shown in Fig 1.

Based on the above analysis, we propose hypothesis H1a.

H1a: Controlling other factors, Digital infrastructure policies exacerbate the financing constraints of Non-SOEs.

### 2.2. Positive mitigating effects of digital infrastructure policies on the Non-SOEs financing constraints

Digital infrastructure constitutes an evolutionary outcome of industrial and technological advancement (Fang et al., 2023) [16], forming a critical subsystem within modern infrastructure architectures. Distinct from mature physical infrastructure networks (e.g., railways, ports), it serves as the foundational pillar for digital economic development (CAICT, 2023), enabling regional digital transformation, enhancing urban technological capabilities, and establishing competitive digital advantages. The accelerating demand for digital infrastructure necessitates strategic governmental planning embodied in rational governance frameworks that coordinate goal-setting, institutional design, and implementation pathways.

The characteristics of government rationality lie in the rational behavior patterns, where the government acts as a rational economic entity, a rational political entity, and a rational contractual entity. Contrary to narrow self-interest models, Chinese local governments prioritize multidimensional development objectives (Lu, 2023) [17] by constraining suboptimal behaviors including regional protectionism, redundant investments, and fiscal maximization strategies. Empirical evidence emerges from policy documents emphasizing *national unified market construction* and interregional coordination, exemplified by strategic initiatives like the *East Data West Computing project* and *Beijing-Shandong Digital Valley*. Through such institutional rationality, governments alleviate digital transformation costs while fostering virtuous cycles of infrastructure investment, fiscal sustainability, and enterprise decision-making optimization.

Digital infrastructure implementation primarily alleviates local fiscal pressures. First, while initial-phase investments demand substantial allocations for technological deployment and regulatory frameworks (Xiang and Zhao, 2023) [18], the interoperable nature of digital systems enables cross-jurisdictional data fluidity that transcends physical infrastructure limitations. This spatial integration minimizes redundant fiscal allocations while liberating human capital (Zhang et al., 2023; Li and Wang, 2020) [19,20]. Second, AI-enhanced fiscal management systems built on digital infrastructure in various regions, integrated with artificial intelligence technology, restrain the soft budget constraint scenarios, effectively prevent, eliminate, and curb the illegal use of budgetary resources, avoid budget resource waste due to overlapping goals at different government levels, and enhance the governance and allocation efficiency of budgetary resources. Third, through credit space expansion and government-backed financing guarantees, these systems alleviate debt accumulation pressures while enhancing financial resource availability for Non-SOEs.

Digital infrastructure enhances fiscal revenue quality. First, intelligent tax administration systems like *Golden Tax III* in China leverage big data analytics to monitor corporate financial activities and tax behaviors across income-expenditure balances, tax liabilities, and inventory flows (Chen et al., 2024; Cai et al., 2021) [8,21]. This technological governance reduces tax evasion while establishing fund supply-demand bridges (Wu et al., 2024; Guedhami and Pittman, 2008) [22,23], alleviating corporate financing constraints (Liu and Li, 2022; Kong and Yuan, 2021) [24,25]. Cai et al. (2021) [8] demonstrated that the tax effect brought about by *Golden Tax Phase III* significantly curbs the tax evasion behavior of Non-SOEs and weakens their internal financing capabilities. Second, digital infrastructure diversifies fiscal revenue and enhances market competition by enabling digital-real economy integration (Xiang and Zhao, 2023; Zhao et al., 2021) [18,26], reducing land finance or traditional industries dependency through emerging digital sectors. Third, digital infrastructure helps optimize local industrial structures. Structural optimization effects manifest through 5G and IoT technologies reshaping industrial value chains, as well as accelerating the upgrading of industrial structures (National Information Center and Huawei, 2020), where innovation-enhancing resource allocation prompts local governments to prioritize credit allocation for Non-SOEs in strategic industries through targeted subsidies and interest rate incentives (Liu and Li, 2022; Che and Xue, 2017) [24,27].

**Fig 2:**
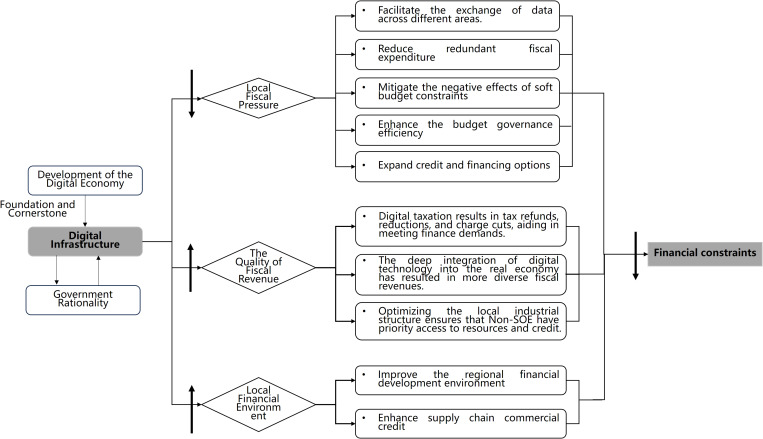
The Positive mitigating effects of digital infrastructure policies on the Non-SOEs financing constraints.

Digital infrastructure elevates regional financial ecosystems. On the one hand, improving the regional financial development levels. algorithm-driven digital finance exhibits inclusive characteristics that particularly benefit resource-constrained SMEs via enhanced credit accessibility (Song et al., 2023; Lang et al., 2021; Wang and Liu, 2021) [28–30]. On the other hand, enhancing commercial credit in the supply chain. The data-driven approach, built on digital infrastructure, facilitates the realization of data penetration, connecting information silos and promoting interconnectivity. It breaks the physical space constraints between organizations within industries, mapping the physical world into a digital space. This strengthens inter-company collaboration, reduces communication and agency costs, and creates favorable conditions for knowledge diffusion and correcting resource misallocation (Qi and Xiao, 2020; World Bank and Development Research Center, 2019) [31,32]. These mechanisms foster interest-aligned commercial networks where accounts payable/receivable function as alternative financing instruments (Qi et al., 2022; Peng et al., 2022; Zhao et al., 2021; Chen and Liu, 2021; Lu and Yang, 2011; Shi and Zhang, 2010; Yu and Pan, 2010) [26,33–38], collectively mitigating enterprise financing constraints. The diagram illustrating the positive effects is shown in Fig 2.

Based on the above analysis, we propose hypothesis H1b.

H1b: Controlling other factors, Digital infrastructure policies alleviate financing constraints of Non-SOEs.

## 3. Research design

### 3.1. Data and samples

In 2014, at the first session of the Central Network Security and Informatization Leading Group, General Secretary Xi Jinping extensively directed the establishment of digital infrastructure. 23 provinces in China have fully embraced the construction of digital infrastructure as a fundamental element for construction. This study focuses on the data of China’s A-share private listed companies from 2015 to 2021 and applies the following methodologies: (1) Companies listed in the financial industry are not included. (2) Companies with the stock codes ST, and *ST are eliminated. (3) Companies with incomplete financial data are excluded, resulting in a final sample size of 10,729 observations. (4) All continuous variables are winsorized at the 1st and 99th percentiles. This paper collects policy textual data on digital infrastructure construction from the websites of provincial and municipal governments and the PKULAW database (which is publicly accessible via its official website: https://www.pkulaw.com/law). Financial data is obtained from the CSMAR (which is publicly accessible via its official website: https://data.csmar.com/) and analyzed using stata17.

### 3.2. Variable measures

**Digital Infrastructure Policies (*Policy*).** In the *Outline of the 14th Five-Year Plan for National Economic and Social Development and Long-Range Objectives Through the Year 2035 of China*, new infrastructure is defined as information infrastructure, integrated infrastructure, and innovation infrastructure. The Digital China Development Report (2021) issued by the National Internet Information Office in 2022 evaluate the development level of digital infrastructure across 31 provinces, regions, and municipalities based on network infrastructure, new computing power systems, and large-scale deployment and application of IPV6. Additionally, the *Overall Layout Plan for Digital China Construction* issued by the State Council in 2023 classifies and integrates the development and construction of digital infrastructure into three aspects: network infrastructure, computing power infrastructure, and application infrastructure. Based on the classification standards of the aforementioned policies and referring to Wang Hai et al. (2023) [1], keywords related to digital infrastructure construction were selected, including digital infrastructure, new infrastructure, network infrastructure, communication infrastructure, computing power infrastructure, intelligent infrastructure, IPV6, 5G, industrial internet, Internet of Things, big data infrastructure, cloud computing, cloud computing platforms, gigabit optical networks, Beidou, and urban intelligent control centers. These keywords were manually searched in policy documents on the websites of provincial and municipal governments and the Peking University Legal Database (PKULAW) (*Considering data omissions, we also searched for policies related to digital infrastructure in the “Digital Economy Policy” database on the China Research Data Services Platform (CNRDS) to supplement the data*). Policies that were clearly unrelated to digital infrastructure construction were identified and excluded. As a result, digital infrastructure policies textual data at the provincial level in China from 2015 to 2021 were obtained.

**Financing constraints (*SA*).** Referring to Hadlock and Pierce (2010) [39], the financing constraints *SA* is constructed according to equation (1). The larger the *SA*, the greater the financing constraints of Non-SOEs.


$$SA_{it}=-0\mathrm{.737}\times Size_{it}+0.043\times Siz{e_{it}}^{2}-0\mathrm{.040}\times Age_{it}$$ (1)

Note: S*ize* is the size of the company, and *Age* is the age of the company since the listing date.

**Moderating variables.** To test the mechanism effect of digital infrastructure policies on alleviating the financing constraints of Non-SOEs, we use the following variables: (1) Local fiscal burdens (*Press*). *Press* = (local fiscal general budget expenditure – local fiscal general budget revenue)/ local fiscal general budget revenue. (2) Local fiscal revenue quality (*Score*). Following Chen and Wang (2023) [40] and considering data availability, the “local fiscal revenue quality (*Score*)” is constructed using an entropy method to synthesize twelve indicators across three dimensions: quantity, structure, and sustainability of fiscal revenue. (3) Overall industrial structure upgrade (*Upgrade*). *Upgrade* = Ratio of primary industry added value to GDP *1 + ratio of secondary industry added value to GDP *2 + ratio of tertiary industry added value to GDP *3. (4) Proportion of tertiary industry in GDP (*Stru3*), measured as tertiary industry output/Regional GDP. (5) Digital Financial Inclusion Index (*Index*), measured by the Digital Financial Inclusion Index released by Peking University. (6) Financial development efficiency (*Finance*), measured by the natural logarithm of regional financial industry value added. (7) Net commercial credit (*NTC*) and the proportion of accounts receivable (*AP*). Referring to Zhang et al. (2012) [41], *NTC* is calculated as the balance of accounts payable, notes payable, and advance receipts minus accounts receivable, notes receivable, and advance payments, divided by total assets. *AP* is calculated as the sum of accounts payable, notes payable, and advance receipts divided by total assets.

We employ the following moderate variables in the heterogeneity tests: (1) Policy Issuer. By manual data collection, we ascertain whether the issuer is the provincial people's government. (2) Company life cycle. Referring to the cash flow model method by Liu et al. (2020) [42], the company lifecycle is divided into growth, maturity, and decline stages based on the combinations of positive and negative operating cash flow, investment cash flow, and financing cash flow. (3) Supply chain support. Following Zeng and Tang (2023) [43], we first identify the main suppliers and customers of Non-SOEs, including both listed and non-listed companies. The ownership of listed companies is obtained from *CSMAR* (https://data.csmar.com/), while the ownership of non-listed companies is manually collected from *Qichacha Website* (Business information search tools in China: https://www.qcc.com/).

**Control variables.** Referring to Wei et al. (2014) [44], Pan et al. (2019) [45], and Wang et al. (2023) [1], we control for the following firm-level variables: firm size (*Size*), asset-liability ratio (*Lev*), return on equity (*ROE*), total asset turnover (*ATO*), the growth rate of operating revenues (*Growth*), share of tangible assets (*Fixratio*), management shareholding (*Mshare*), shareholding of the largest shareholder (*Top1*), duality of CEO and Board Chairman (*Dual*), board size (*Board*), the proportion of independent directors (*Indep*). The variables at the regional level are regional gross domestic product (*GDP*), regional size (*Areasize*), regional tertiary industry share (*Stru3*), regional spending Index (*Areaabli*), and regional scientific and technological Index (*Areatech*). In addition, this paper also considers year and industry-fixed effects (*Year/Industry Fixed Effect*) and area-fixed effects (*Area Fixed Effect*). (See Table 1 for details).

**Table 1:** Variable definitions.

Category / Variable		Description
**Financing constraints**	*SA*	According to the *SA* calculated by Hadlock et al. (2010), a higher *SA* indicates more severe financing constraints.
**Digital Infrastructure Policies**	*Policy*	The number of digital infrastructure policy documents in provinces
**Firm Characteristics**		
Firm Size	*Size*	The natural log of a firm's total assets
Debt-to-Asset Ratio	*Lev*	Total liability scaled by total assets
Return on Equity	*ROE*	Income before extraordinary items divided by Equity
Total Asset Turnover Operating income	*ATO*	Operating income scaled by Total Asset
The Growth rate of Operating Revenue	*Growth*	Operating income_t_ scaled by Operating income_t-1_–1
Tangible Assets	*Fixratio*	The proportion of tangible assets to total assets
Management’s Shareholdings	*Mshare*	Management’s shareholdings scaled by total equity
The Largest Shareholder’s Shareholdings	*Top1*	The proportion of the largest shareholder's shareholdings
CEO duality	*Dual*	Equals 1 if CEO is the chairman of the board, and 0 otherwise
Board Size	*Board*	The natural log of the number of board members
Proportion of Independent Directors	*Indep*	The proportion of independent directors on the board of directors
**Region Characteristics**		
Gross Domestic Product	*GDP*	The natural log of the GDP in the region where the firm is located
Regional Size	*Areasize*	The natural log of the number of prefecture-level cities in the region where the firm is located
Proportion of the Regional Tertiary Industry	*Stru3*	tertiary industry output scaled by Regional GDP
Regional Expenditure Capacity	*Areaabli*	The natural log of the general budget expenditure in the region where the firm is located
Regional Technological Capabilities	*Areatech*	The natural log of scientific and technological expenditures where the firm is located
	*Year Fixed Effect*	Year Fixed Effect
	*Industry Fixed Effect*	Industry Fixed Effect
	*Area Fixed Effect*	Eight Major Geographical Zones of China: East China, South China, North China, Central China, Southwest Region, Northwest Region, Northeast Region, Hong Kong, Macau, and Taiwan

### 3.3. Regression models

Based on the above analysis, we construct Model (2) to test Hypothesis 1, which examines the relationship between digital infrastructure policies and the financing constraints of Non-SOEs. Economically, this model incorporates year, industry, and area fixed effects to isolate the net impact of digital infrastructure policies (*Policy*) on financing constraints (measured by the *SA*), controlling for time-varying macro trends (e.g., national policies), industry-specific characteristics (e.g., sectoral financing needs), and regional heterogeneities (e.g., institutional environments). The inclusion of firm-level controls (e.g., size, leverage, growth) ensures that the estimated coefficient *β*_1_ reflects the policy's direct effect rather than confounding factors. A statistically significant negative *β*_1_ would indicate that increasing digital infrastructure policy intensity alleviates financing constraints of Non-SOEs, aligning with the theoretical hypothesis that such policies optimize fiscal resources, enhance financial inclusion, and reduce information asymmetry. This model thus serves as a critical tool to quantify the policy's economic efficacy and validate the study’s core argument about the micro-level benefits of digital infrastructure through macro-institutional channels.


$$SA_{it}=\beta_{0}+\beta_{1}Policy_{it}+\sum \beta_{j}Controls_{jit}+\sum {\mathrm{\backslash nolimits}}_{t}\alpha_{t}Year{\mathrm{\backslash nolimits}}_{t}+\sum {\mathrm{\backslash nolimits}}_{i}\gamma {\mathrm{\backslash nolimits}}_{i}Industry{\mathrm{\backslash nolimits}}_{i}+\sum {\mathrm{\backslash nolimits}}_{i}\lambda {\mathrm{\backslash nolimits}}_{i}Area{\mathrm{\backslash nolimits}}_{i}+\varepsilon_{it}$$ (2)

## 4. Empirical results

### 4.1. Descriptive statistics

According to the descriptive statistics of the main variables (see Table 2 for details), the mean value of the *Policy* is 1.309, with a maximum value of 11. This indicates that from 2015 to 2021, the provincial regions where Non-SOEs are located have issued an average of 1.31 digital infrastructure policies, with a maximum of 11 policies. The mean and standard deviation of the firm *SA* is −3.837 and 0.224, indicating variation in the degree of financing constraints for Non-SOEs. The mean of *SA* is slightly less than but close to the median, and the sample distribution is slightly left-skewed. Values above the median for digital infrastructure policy introductions within the same year are categorized as the “High Policy Group (*HighPo*)” for univariate tests. Table 3 presents the results of this univariate test. When *HighPo* is set to 1, indicating provinces with a higher number of digital infrastructure policies, the mean and median of the Non-SOEs’ *SA* are −3.852 and −3.838, respectively. These values are significantly lower at the 10% and 1% levels compared to the *SA* of Non-SOEs in provinces with fewer digital infrastructure policies.

**Table 2:** Descriptive statistics.

Variables	N	Mean	Max	Min	Std. Dev.
*Policy*	10729	1.309	11.000	0.000	2.138
*SA*	10729	−3.837	−3.341	−4.428	0.224
*Size*	10729	22.021	25.654	19.956	1.094
*Lev*	10729	0.391	0.882	0.059	0.187
*ROE*	10729	0.064	0.384	−0.799	0.147
*ATO*	10729	0.624	2.361	0.074	0.382
*Growth*	10729	0.200	2.559	−0.570	0.422
*Fixratio*	10729	0.910	1.000	0.507	0.100
*Mshare*	10729	19.393	68.399	0.000	20.264
*Dual*	10729	0.375	1.000	0.000	0.484
*Top1*	10729	31.032	68.710	7.920	13.335
*Board*	10729	8.109	12.000	5.000	1.432
*Indep*	10729	37.953	57.140	33.330	5.308
*GDP*	10729	10.888	11.731	8.217	0.695
*Areasize*	10729	2.604	3.045	1.386	0.353
*Stru3*	10729	51.209	61.496	40.201	3.856
*Areaabli*	10729	9.124	29.812	7.525	0.473
*Areatech*	10729	5.682	7.064	2.711	1.018

**Table 3:** Univariate test.

Variable	*HighPo* = 1			*HighPo* = 0			Mean Difference	Median Difference
	N	Mean	Median	N	Mean	Median		
*SA*	3496	−3.852	−3.838	7233	−3.830	−3.823	−0.022^*^ (1.862)	−0.0015^***^ (16.917)

Table 3 presents the results of univariate test. When *HighPo* is set to 1, indicating provinces with a higher number of digital infrastructure policies. The superscripts ***, **, and * indicates statistical significance at 1%, 5% and 10% levels, respectively. We use a robust estimation and report t-values below the coefficients in mean difference and median difference.

### 4.2. Main results

To empirically validate hypothesis H1, we performed regression analyses using Model (2), with Table 4 demonstrating statistically significant negative associations between digital infrastructure policy intensity and Non-SOEs financing constraints (p < 0.01^***^), robust to industry-year-region fixed effects and extended covariate adjustments. To ensure the robustness of the regression results, we further examine the impact of omitted variables and other unobserved factors on the regression results in the robustness tests.

**Table 4:** Panel A. Main Results: Digital Infrastructure Policies and Financing constraints (OLS) and Panel B: Main Results: Digital Infrastructure Policies and Financing constraints (DML).

	(1)	(2)	(3)	
	*SA*	*SA*	*SA*	
*Policy*	−0.012^***^	−0.014^***^	−0.003^***^	
	(−11.501)	(−14.332)	(−2.770)	
*Size*			−0.005	
			(−0.932)	
*Lev*			0.017	
			(0.647)	
*ROE*			0.001	
			(0.043)	
*ATO*			−0.002	
			(−0.139)	
*Growth*			0.021^***^	
			(3.699)	
*Fixratio*			0.109^***^	
			(2.599)	
*Mshare*			0.002^***^	
			(7.394)	
*Dual*			0.036^***^	
			(4.347)	
*Top1*			0.001^**^	
			(2.150)	
*Board*			−0.010^**^	
			(−2.382)	
*Indep*			−0.001	
			(−1.091)	
*GDP*			−0.009	
			(−0.316)	
*Areasize*			−0.004	
			(−0.145)	
*Stru3*			0.003^**^	
			(2.086)	
*Areaabli*			0.063	
			*(1.278)*	
*Areatech*			*−0.016*	
			*(−1.171)*	
*Year & Industry & Area*	*NO*	*Yes*	*Yes*	
Constant	−3.822^***^ (−753.451)	−3.900^***^ (−110.054)	−4.261^***^ (−17.248)	
Observations	10729	10729	10729	
*R* ^*2*^ *_adj*	0.013	0.047	0.135	
	**Linear DML**			
	**Random Forest**	**Lassocv**	**Gradboost**	**Nnet**
	**(1)**	**(2)**	**(3)**	**(4)**
	*SA*	*SA*	*SA*	*SA*
*Policy*	−0.028* (−1.796)	−0.010*** (−9.191)	−0.057*** (−8.480)	−0.004*** (−2.974)
*Controls*	YES	YES	YES	YES
Constant	0.002 (1.256)	−0.000 (−0.082)	0.000 (0.015)	0.000 (0.026)
*Year & Industry & Area*	YES	YES	YES	YES
Observations	10729	10729	10729	10729

Table 4 presents the impact of digital infrastructure Policies on financing constraints of Non-SOEs. All variables are defined in Table 1. The superscripts ***, **, and * indicates statistical significance at 1%, 5% and 10% levels (two-tailed), respectively. We use a robust estimation and report t-values below the coefficients. Data for *Policy* manually collected from the websites of provincial and municipal governments and the PKULAW database. Financial data is obtained from the CSMAR. All variables are winsorized at the 1st and 99th percentiles.

Referring to Bodory et al. (2022) [46], we employ Double Machine Learning (DML) approach to address limitations of conventional causal inference models in handling high-dimensional covariates and nonlinear interaction effects. The DML integrates machine learning algorithms (Random Forest, Lassocv, Gradboost, Nnet) to automatically capture complex data patterns, thereby mitigating model misspecification risks. Implementation involves five key steps: (1) partitioning baseline variables into core controls and fixed effects; (2) conducting 3-fold cross-validation for sample subdivision; (3) training multiple ML models to regress treatment/outcome variables on covariates; (4) generating residuals through dual prediction; (5) estimating causal effects via linear regression of outcome residuals on treatment residuals. The final estimator solves:


$$\hat{SA}=g(Controls),\hat{Policy}=m(Controls)$$ (3)

$$\tilde{SA}=SA-\hat{SA},\;\hat{Policy}=Policy-\hat{Policy}$$ (4)

$$\hat{SA}=\theta \hat{Policy}+\epsilon$$ (5)

Equation (3) estimates fitted values for *SA* and *Policy* conditional on observed covariates, while Equation (4) extracts orthogonalized residuals through partial correlation analysis. Equation (5) then calculates the debiased causal parameter *θ* via residual-on-residual regression, with Table 4 Panel B confirming persistent statistical significance across year-industry-region fixed effects specifications. Notably, covariate-adjusted models (Columns 1–4) persistently demonstrate statistically significant negative coefficients (p < 0.01), empirically validating digital infrastructure policies’ constraint-alleviation effects on Non-SOEs.

Table 4 Panel A and Panel B collectively offer comprehensive economic insights into the impact of digital infrastructure policies on Non-SOEs’ financing constraints. Panel A utilizes ordinary least squares (OLS) regression, revealing a statistically significant negative relationship between policy intensity and the *SA* (financing constraints), with coefficients ranging from −0.012 to −0.003 (all p < 0.01). This indicates that each additional digital infrastructure policy is associated with a measurable reduction in financing constraints, even after controlling for year-fixed effects, industry-fixed effects, and area-fixed effects. The inclusion of firm-level controls ensures that the observed effect is not confounded by firm-specific attributes, reinforcing the causal interpretation that policy implementation drives constraint alleviation.

Panel B employs DML, a robust nonparametric method designed to handle high-dimensional data and nonlinear interactions. Across diverse algorithms, Panel B consistently reports negative coefficients for the policy variable (ranging from −0.057 to −0.004, all p < 0.05), aligning with Panel A’s findings. This nonparametric validation is critical for addressing model misspecification risks, as DML automatically captures complex data patterns without imposing strict parametric assumptions.

Together, these panels provide convergent evidence from both traditional parametric and advanced nonparametric methodologies, confirming that digital infrastructure policies systematically reduce Non-SOEs’ financing constraints. The economic significance lies in their demonstration of a reliable, policy-driven mechanism to enhance private enterprises’ access to finance, with implications for optimizing digital transformation strategies to foster fiscal sustainability and microeconomic vitality.

### 4.3. Robustness test

Digital infrastructure is an exogenous variable that may exhibit reciprocal causality and endogeneity concerns. Specifically, the significant statistical relationship of financing constraints could potentially originate from endogenous biases such as omitted variables. To ensure the robustness of the regression results, we consequently implement four methods for endogeneity robustness tests.

#### 4.3.1. Instrumental Variable Test (IV).

While policy is an exogenous variable, endogeneity concerns related to reciprocal causality cannot be ruled out. For instance, regions characterized by fewer financial constraints on private enterprises typically exhibit elevated levels of marketization and economic development, thereby facilitating the implementation of digital infrastructure policies. To address this potential bias, we selectively employ three instrumental variables: the industrial solid waste comprehensive utilization rate (*Environment*), the natural logarithm of industrial pollution control investment completion amounts (*Indupollution*), and the natural logarithm of daily urban sewage treatment capacity (*Sewage*)(*Some city data is missing, resulting in a loss of the overall regression sample*).

Table 5 shows the IV-2SLS regression results using the instrumental variable. Column (1) in Table 5 presents the first-stage regression results, and the *Envirct*, *Indupollution*, *Sewage*, and *Policy* are significantly and positively correlated. Column (2) displays the regression coefficient of digital infrastructure policies on financing constraints for Non-SOEs after excluding endogeneity interference, which is −0.058 and significant at the 5% level. This indicates that digital infrastructure policies have a significant suppressive effect on financing constraints. The instrumental variable estimation results further demonstrate that the conclusions of this study are robust.

**Table 5:** IV test.

	The First Stage	The Second Stage
	*Policy*	*SA*
	(1)	(2)
*Policy*		−0.058^**^
		(−1.988)
*Envirct*	0.009^***^	
	(6.145)	
*Indupollution*	0.636^***^	
	(5.542)	
*Sewage*	0.166^***^	
	(4.996)	
Constant	−2.750^***^	−4.688^***^
	(−3.208)	(−14.467)
Controls	Yes	Yes
*Year & Industry & Area*	Yes	Yes
*R* ^2^ *_adj*	0.461	0.087
Observations	7543	7543
Identification test of IV	Kleibergen-Paap rk LM:270.127 Cragg-Donald Wald F:84.123	

Table 5 presents the IV test. The superscripts ***, **, and * indicate statistical significance at 1%, 5% and 10% levels (two-tailed), respectively. We use a robust estimation and report t-values below the coefficients. We employ the comprehensive utilization rate of industrial solid waste (*Environment*), the natural logarithm of completed investments in industrial pollution control (*Indupollution*), and the natural logarithm of urban sewage treatment capacity per day (*Sewage*) as instrumental variables.

#### 4.3.2. Heckman’s Two-Stage test.

To address endogeneity issues arising from sample selection bias, we employ Heckman’s two-stage model for testing. Columns (1), (2), and (3) of Table 6 present the results of the first-stage regressions, where the instrumental variables *Envirct*, *Sewage*, and *Indupollution* are significantly and positively associated with the high policy group (*HighPo*) (*The high policy group (HighPo) is defined in the same way as in the univariate analysis, which is whether the province’s digital infrastructure policy is above the median value for the same year*). The regression results in columns (4), (5), and (6) indicate that, after accounting for endogeneity due to sample selection bias, digital infrastructure policies significantly alleviate Non-SOEs financing constraints, which is consistent with the main regression results.

**Table 6:** Heckman’s two-stage test.

	(1) The First Stage	(2) The First Stage	(3) The First Stage	(4) The Second Stage	(5) The Second Stage	(6) The Second Stage
	*HighPo*	*HighPo*	*HighPo*	*SA*	*SA*	*SA*
*Envirct*	0.016^***^					
	(4.605)					
*Sewage*		3.395^***^				
		(20.663)				
*Indupollution*			0.103^***^			
			(3.162)			
*Policy*				−0.011^***^	−0.010^***^	−0.011^***^
				(−4.185)	(−8.700)	(−9.687)
				0.064^***^	0.011	0.041^***^
IMR				(9.500)	(1.401)	(4.974)
Constant	−15.514^***^	−0.948	−16.381^***^	−4.035^***^	−3.336^***^	−3.787^***^
	(−10.294)	(−0.726)	(−14.995)	(−13.062)	(−12.574)	(−13.932)
Controls	Yes	Yes	Yes	Yes	Yes	Yes
*Year & Industry & Area*	Yes	Yes	Yes	Yes	Yes	Yes
*R* ^2^ *_adj*	0.501	0.379	0.341	0.088	0.078	0.080
Observations	7543	7543	7543	7543	7543	7543

Table 6 presents the Heckman’s Two-Stage test. The superscripts ***, **, and * indicate statistical significance at 1%, 5% and 10% levels (two-tailed), respectively. We use a robust estimation and report t-values below the coefficients. We employ the *Envirct, Sewage, and Indupollution* as instrumental variables.

#### 4.3.3. Keep only samples of digital infrastructure policies that have changed.

In the context of increasing changes in digital infrastructure policies, if these changes alleviate firms’ financing constraints, it suggests that the policy is effective in mitigating these constraints, and the endogenous influence can be somewhat ruled out. The regression results for the sample of 7,066 firm-years are presented in Table 7. The *Policy* coefficient in column (1) of Table 7 is significantly positive at the 1% level, indicating that increases in digital infrastructure policies can alleviate the financing constraints faced by private listed companies. Column (2) shows the mean values before and after the sample change, specifically the moving averages *avgSA* and *avgPolicy*. The moving average *avgPolicy* of digital infrastructure policies is significantly negatively correlated with the moving average of the financing constraints (*SA*) at the 1% level. These results are consistent with the main results above.

**Table 7:** Keep only samples of digital infrastructure policies that have changed.

	(1)	( 2)
	*SA*	*avgSA*
*Policy*	−0.005^***^	
	(−4.253)	
*avgPolicy*		−0.007^***^
		(−4.761)
Constant	−4.220^***^	−4.285^***^
	(−15.970)	(−18.418)
Controls	Yes	Yes
*Year & Industry & Area*	Yes	Yes
*R* ^2^ *_adj*	0.150	0.099
Observations	7066	7066

Table 7 presents the test which keeps only samples of digital infrastructure policies which have changed. The superscripts ***, **, and * indicate statistical significance at 1%, 5% and 10% levels (two-tailed), respectively. We use a robust estimation and report t-values below the coefficients.

#### 4.3.4. Other robustness tests.

① Change the alternative variables of financing constraints. The results were re-regressed by drawing on the *FC* constructed by the methods of Hdlock and Pierce (2010) [39], Kuang (2010) [47], and others. ② Add other control variables that affect financing constraints: book-to-market ratio (*BM*), and institutional investor shareholding (*INST*). ③ Regression is performed by using the lag term. The results in Table 8 show that the conclusions are still robust.

**Table 8:** Other robust tests.

	(1)	(2)	(3)	(4)
	*FC*	*FC*	*SA*	*SA*
*Policy*	−0.001^*^		−0.003^***^	
	(−1.669)		(−2.891)	
*L1.Policy*		−0.004^**^		−0.0054^*^
		(−2.260)		(−1.733)
*BM*			−0.085^***^	
			(−3.907)	
*INST*			0.001^***^	
			(3.509)	
Controls	Yes	Yes	Yes	Yes
Constant	4.772^***^	5.011^***^	−4.421^***^	−4.661^***^
	(42.397)	(42.949)	(−17.673)	(−17.485)
*Year & Industry & Area*	Yes	Yes	Yes	Yes
*R* ^2^ *_adj*	0.780	0.806	0.145	0.098
Observations	10729	6292	10729	6292

Table 8 presents the other robust tests. The superscripts ***, **, and * indicate statistical significance at 1%, 5% and 10% levels (two-tailed), respectively. We use a robust estimation and report t-values below the coefficients.

### 4.4. Mechanism tests

To avoid reverse causality disrupting the test results, we adopt the two-stage method from Di and Laux (2022) [48], and Ma and Chen (2022) [49] to test the mechanism effects, as shown in Model (6).


$$\begin{array}{c} X_{it}=\beta_{0}+\beta_{1}Policy_{it}+\sum \beta_{j}Controls_{jit}+\sum {\mathrm{\backslash nolimits}}_{t}\alpha_{t}Year_{t}+\sum {\mathrm{\backslash nolimits}}_{i}\gamma_{i}Industry_{i}+\sum {\mathrm{\backslash nolimits}}_{i}\lambda {\mathrm{\backslash nolimits}}_{i}Area{\mathrm{\backslash nolimits}}_{i}+\varepsilon_{it}\\ SA_{it}=\beta_{0}+\beta_{1}{\hat{M}}_{it}+\sum \beta_{j}Controls_{jit}+\sum {\mathrm{\backslash nolimits}}_{t}\alpha {\mathrm{\backslash nolimits}}_{t}Year{\mathrm{\backslash nolimits}}_{t}+\sum {\mathrm{\backslash nolimits}}_{i}\gamma {\mathrm{\backslash nolimits}}_{i}Industry{\mathrm{\backslash nolimits}}_{i}+\sum {\mathrm{\backslash nolimits}}_{i}\lambda {\mathrm{\backslash nolimits}}_{i}Area{\mathrm{\backslash nolimits}}_{i}+\varepsilon_{it} \end{array}$$ (6)

The first equation X is the mechanism variables for the mechanism effect test, including local financial pressure (*Press*); the substitute variables for measuring the quality of local financial revenue are the composite index calculated by the entropy method (*Score*), the overall upgrading of the industrial structure (*Upgrade*), and the proportion of the tertiary industry in GDP (*Stru3*); the substitute variables in terms of the regional financial levels are: the Digital Inclusive Finance Index (*Index*), and the efficiency of financial development (*Finance*); and the substitute variables for measuring the commercial credit are: the Net Commercial Credit (*NTC*), and the accounts payable ratio (*AP*). The second equation incorporates the predictive mechanism variable $\hat{M}$ into the model to regress the *SA*. Tables 9–11 report the results of the mechanism test results.

**Table 9:** Mechanism tests: From the perspective of alleviating fiscal burdens.

	(1)	(2)
	*Press*	*SA*
*Policy*	−0.025^***^	
	(−16.189)	
$\hat{P}ress$		**0.124** ^**^
		**(2.568)**
Controls	Yes	Yes
Constant	4.953^***^	−4.877^***^
	(18.917)	(−17.489)
*Year & Industry & Area*	Yes	Yes
*R* ^2^ *_adj*	0.902	0.135
Observations	10729	10729

Table 9 presents the mechanism tests from the perspective of alleviating fiscal burdens. The superscripts ***, **, and * indicate statistical significance at 1%, 5% and 10% levels (two-tailed), respectively. We use a robust estimation and report t-values below the coefficients.

**Table 10:** Mechanism tests: From the Perspective of improving the quality of fiscal revenue.

	(1)	(2)	(3)	(4)	(5)	(6)
	*Score*	*SA*	*Upgrade*	*SA*	*Stru3*	*SA*
*Policy*	0.001^***^		0.001^***^		0.250^***^	
	(9.594)		(8.592)		(20.797)	
$\hat{S}core$		**-5.563** ^**^				
		**(−2.568)**				
$\hat{U}pgrade$				**-3.145** ^***^		
				**(−2.770)**		
$\hat{S}tru3$						**-0.009** ^**^
						**(−1.971)**
Controls	Yes	Yes	Yes	Yes	Yes	Yes
Constant	0.067^***^	−3.890^***^	1.816^***^	1.451	42.719^***^	−3.728^***^
	(5.901)	(−22.196)	(68.121)	(0.710)	(13.616)	(−12.719)
*Year & Industry & Area*	Yes	Yes	Yes	Yes	Yes	Yes
*R* ^2^ *_adj*	0.830	0.135	0.934	0.135	0.651	0.134
Observations	10729	10729	10729	10729	10729	10729

Table 10 presents the mechanism tests from the perspective of improving the quality of fiscal revenue. The superscripts ***, **, and * indicate statistical significance at 1%, 5% and 10% levels (two-tailed), respectively. We use a robust estimation and report t-values below the coefficients.

**Table 11:** Mechanism tests: From the Perspective of improving regional financial levels.

	(1)	(2)	(3)	(4)	(5)	(6)	(7)	(8)
	*Finance*	*SA*	*Index*	*SA*	*NTC*	*SA*	*AP*	*SA*
*Policy*	0.002^***^		1.991^***^		0.002^***^		0.001^**^	
	(7.000)		(37.133)		(4.120)		(2.480)	
$\hat{F}inance$		**-1.305** ^***^						
		**(-2.770)**						
$\hat{I}ndex$				**-0.002** ^***^				
				**(-2.770)**				
$\hat{N}TC$						**-1.278** ^***^		
						**(-2.770)**		
$\hat{A}P$								**-2.913** ^***^
								**(-2.770)**
Controls	Yes	Yes	Yes	Yes	Yes	Yes	Yes	Yes
Constant	2.155^***^	−1.447	324.180^***^	−3.752^***^	−0.326^***^	−4.677^***^	−0.124	−4.623^***^
	(34.878)	(−1.428)	(33.089)	(−13.038)	(−2.688)	(−15.307)	(−1.437)	(−15.712)
*Year & Industry & Area*	Yes	Yes	Yes	Yes	Yes	Yes	Yes	Yes
*R* ^2^ *_adj*	0.842	0.135	0.974	0.135	0.194	0.135	0.410	0.135
Observations	10729	10729	10729	10729	10729	10729	10729	10729

Table 11 presents the mechanism tests from the perspective of improving regional financial levels. The superscripts ***, **, and * indicate statistical significance at 1%, 5% and 10% levels (two-tailed), respectively. We use a robust estimation and report t-values below the coefficients.

Table 9 reports the results of the mechanism test for alleviating fiscal burdens. Column (1) shows that the regression coefficient of *Policy* in the first stage is significantly negative, indicating that an increase in digital infrastructure policies corresponds to lower fiscal burdens. In other words, digital infrastructure alleviates fiscal burdens, which is consistent with expectations. Column (2) shows that the regression coefficient of$\hat{P}ress$ in the second stage is significantly positive, indicating that changes in fiscal burdens and Non-SOEs’ financing constraints move in the same direction. If fiscal burdens decrease, the financing constraints of Non-SOEs also decrease. The results in Table 9 indicates that alleviating fiscal burdens is one of the important mechanisms through which digital infrastructure policies alleviate financing constraints for Non-SOEs.

Digital infrastructure policies alleviate Non-SOEs’ financing constraints by reducing local fiscal burdens. As shown in Table 9, a one-unit increase in policy intensity significantly decreases fiscal burden (Press) by 0.025 units (p < 0.01), while a reduction in fiscal burden is associated with a 0.124-unit decrease in financing constraints (*SA*, p < 0.05). This indicates that digital infrastructure enhances fiscal governance efficiency through cross-jurisdictional data integration and AI-powered fiscal management systems (e.g., Golden Tax III), reducing redundant expenditures and curbing soft budget constraints.

Table 10 reports the results of the mechanism test for improving the quality of fiscal revenue. Columns (1), (3), and (5) show that the regression coefficients of *Policy* in the first stage are significantly positive, indicating that the more digital infrastructure policies there are, the higher the fiscal revenue quality, the greater the overall industrial upgrading degree, and the higher the proportion of the tertiary industry. In other words, digital infrastructure has improved the fiscal revenue quality and stimulated overall industrial upgrading, consistent with expectations. Columns (2), (4), and (6) show that the regression coefficients of $\hat{S}core$, $\hat{U}pgrade$, $\;\hat{S}tru3$ in the second stage are significantly negative, indicating that the higher the fiscal revenue quality, the greater the overall industrial upgrading and the higher the proportion of the tertiary industry, the more it can alleviate the financing constraints of Non-SOEs. The results of Table 10 show that improving the quality of fiscal revenue is one of the important paths for digital infrastructure policies to alleviate the financing constraints of Non-SOEs.

Digital infrastructure policies enhance fiscal revenue quality, thereby alleviating financing constraints. The results show positive associations between policy intensity and fiscal revenue quality (*Score*, *β* = 0.001, p < 0.01), industrial upgrading (*Upgrade*, *β* = 0.001, p < 0.01), and the tertiary industry share (*Stru3*, *β* = 0.250, p < 0.01). Higher fiscal revenue quality and industrial sophistication significantly reduce *SA* (*Score β* = −5.563, p < 0.01). This mechanism operates through digital-real economy integration, which diversifies revenue sources (digital industry taxes) and reduces dependence on land finance.

Table 11 reports the results of the mechanism tests for improving regional financial levels. Columns (1), (3), (5), and (7) show that the regression coefficients of *Policy* in the first stage are significantly positive. This indicates that regions with more digital infrastructure policies have higher digital inclusive finance indices, greater financial development efficiency, higher net commercial credit, and higher accounts payable ratios. In other words, digital infrastructure improves regional financial development levels and enhances corporate commercial credit, consistent with expectations. Columns (2), (4), (6), and (8) show that the regression coefficients of $\hat{F}inance$, $\hat{I}ndex$, $\hat{N}TC$, $\hat{A}P$ in the second stage are significantly negative. This suggests that the higher the regional digital inclusive finance index, the greater the financial development efficiency, the higher the net commercial credit, and the higher the accounts payable ratio, the more able they are to alleviate the financing constraints of Non-SOEs, which is also consistent with expectations. The results in Table 11 indicate that improving the regional financial levels is one of the important mechanisms through which digital infrastructure policies alleviate financing constraints for Non-SOEs.

Digital infrastructure policies improve regional financial development and supply chain commercial credit to alleviate financing constraints. A one-unit policy increase correlates with significant improvements in digital financial inclusion (*Index*, *β* = 1.991, p < 0.01), financial efficiency (*Finance*, *β* = 0.002, p < 0.01), net commercial credit (*NTC*, *β* = 0.002, p < 0.01), and accounts payable ratios (*AP*, *β* = 0.001, p < 0.01), all of which reduce *SA* (*Index β* = −0.002, p < 0.01). Digital platforms facilitate supply chain data integration, enabling SMEs to leverage commercial credit (accounts payable) as alternative financing tools.

### 4.5. Heterogeneity tests

#### 4.5.1. The impact differences between issuing institutions.

Policy efficacy fundamentally depends on institutional coordination between legislative and executive entities (Wang, 2006) [50], a principle particularly applicable to digital infrastructure initiatives whose implementation dynamics are mediated by multilevel promulgating authorities that critically shape enforcement intensity and regional enterprise impacts. Most digital infrastructure policies are formulated and issued by people’s governments, though some are undertaken by departments outside of the people’s government, such as provincial development and reform commissions and provincial economic and information commissions. To empirically disentangle these hierarchical effects, we conducted stratified regression analysis categorizing policies by provincial government authorship status (Table 12). Robustness of intergroup comparisons was ensured through 500-replication bootstrap parametric equality tests, with empirical bootstrapping procedures yielding statistically distinguishable outcomes (p < 0.01) that confirm superior constraint-alleviation effects for government-authored policies. As the highest local administrative authority, the people’s government possesses more comprehensive administrative power and can integrate information and resources from relevant executive agencies in finance, economy, livelihood, education, and other areas. Thus, the policies they formulate consider a broader range and coverage, resulting in stronger implementation and enforcement and a greater impact on enterprises compared to other agencies.

**Table 12:** Heterogeneity Test of the impact differences between issuing institutions.

	(1) Policies issued by people’s governments	(2) Policies issued by other institutions
	*SA*	*SA*
*Policy*	−0.012^***^	−0.005
	(−3.317)	(−1.488)
Controls	Yes	Yes
Constant	−5.110^***^	−4.230^***^
	(−8.889)	(−16.692)
*Year & Industry & Area*	Yes	Yes
Empirical P-value	−0.007^***^	
*R* ^2^ *_adj*	0.136	0.138
Observations	2223	8506

Table 12 presents the heterogeneity test of the impact differences between issuing institutions. The superscripts ***, **, and * indicate statistical significance at 1%, 5% and 10% levels (two-tailed), respectively. We use a robust estimation and report t-values below the coefficients.

#### 4.5.2. The impact differences of life cycles.

The life cycle of a business is a concentrated reflection of its resource collection, comprehensively reflecting the different growth needs of businesses (Dickinson, 2011) [51]. Therefore, at different stages of the life cycle, there are significant differences in the characteristics, resource capabilities, financing needs, and degree of digitalization of businesses (Abernathy et al., 1973; Adizes et al., 1989; Liu and Chen, 2003) [52–54].

In the growth stage, enterprises confront structural capital inadequacies stemming from immature revenue-generation frameworks and endogenous liquidity constraints, compounded by nascent market credibility that amplifies external financing needs (Huang et al., 2016; Tang et al., 2022) [55,56]. Digital infrastructure policies can effectively improve regional finances and stimulate the release of substantial financial resources. Intelligent financial technology can help businesses utilize digital technology to search for data information, open financing channels, and fill the funding gaps during the growth stage.

In the maturity stage, enterprises have relatively stable profitability and market conditions, which can attract many investors to obtain stable external funds, thereby alleviating financing constraints (Tang et al., 2022) [56]. Consequently, the reliance on the digital benefits brought by digital infrastructure policies is low, and thus these policies have almost no impact on the financing constraints of mature businesses.

In the decline stage, most of the market is monopolized by competitors, and the financial situation gradually deteriorates. It is crucial to achieve business transformation to make up for losses; otherwise, the business will face “elimination.” In the digital economy era, businesses urgently need to undergo digital transformation, deeply integrating with digital technology from supply, sales, and finance to adapt to changes in business models, alleviating operational difficulties, and obtaining new capital allocations. Businesses in the decline stage can leverage the digital infrastructure provided by the government to undergo digital transformation, which can regain investor favor. Therefore, businesses in the decline stage can improve financing constraints through digital infrastructure policies.

Referencing the cash flow model by Liu et al. (2020) [42], the company lifecycle is divided into growth, maturity, and decline stage based on the positive and negative combinations of operating cash flow net amount, investment cash flow net amount, and financing cash flow net amount. The regression results are shown in Table 13. In the growth and decline stage, where financing needs are strong, but financing conditions are poor, digital infrastructure policies (*Policy*) have more significantly alleviated corporate financing constraints. To ensure the robustness of the results when comparing coefficients between groups in regression, a “*Bootstrap*” method was used to test the differences in coefficients between groups, with empirical P-values showing significant differences between the growth-maturity and decline-maturity stages.

**Table 13:** Heterogeneity test of the impact differences between life cycles.

	(1) Growth Stage	(2) Maturity Stage	(3) Decline Stage
	*SA*	*SA*	*SA*
*Policy*	−0.005^***^	0.002	−0.006^***^
	(−3.028)	(0.859)	(−2.754)
Controls	Yes	Yes	Yes
Constant	−5.006^***^	−3.485^***^	−3.736^***^
	(−17.391)	(−10.941)	(−11.281)
*Year & Industry & Area*	Yes	Yes	Yes
Empirical P-value	Comparison between (1) and (2) −0.006^**^	Comparison between (3) and (2) −0.007^***^	
*R* ^2^ *_adj*	0.125	0.158	0.204
Observations	4700	3232	2797

Table 13 presents the heterogeneity test of the impact differences between life cycles. The superscripts ***, **, and * indicate statistical significance at 1%, 5% and 10% levels (two-tailed), respectively. We use a robust estimation and report t-values below the coefficients.

#### 4.5.3. The impact differences of supply chain support.

Corporate operational networks exhibit tight interlinkages with supply chain partners (Zeng and Tang, 2023) [43], where state-owned enterprises (SOEs) historically serve as critical stabilizing agents (Liu, 2001; Guo and Ma, 2019; Ye and Luo, 2022) [57–59]. Contrary to resource competition assumptions, recent scholarship demonstrates SOEs’ supply chain participation not only avoids crowding-out effects but actively enhances non-SOEs’ operational capacities through collaborative value creation (Lin et al., 2022) [60]. This aligns with central government mandates articulated by the State Council and SASAC emphasizing SOEs’ pivotal role in post-crisis economic revitalization through supply chain leadership that stimulates cross-ownership synergies. Empirical evidence reveals non-SOEs with SOE supply chain affiliations maintain superior operating cash flow positions relative to peer firms lacking such institutional connections (Zeng and Tang, 2023) [43]. Consequently, non-SOEs operating outside SOE-anchored supply chains demonstrate heightened sensitivity to digital infrastructure policy interventions in alleviating financing constraints, necessitating augmented macroeconomic policy support to compensate for deficient micro-level supply chain stimulation mechanisms. Regression analyses stratified by SOE(Referencing to Zeng and Tang (2023) [43], the major suppliers and customers of Non-SOEs were identified, including both listed and non-listed companies. The ownership of listed companies was obtained from CSMAR, while the ownership of non-listed companies was manually collected from Qichacha). dominance in supply chain roles yield critical insights (Table 14). Columns (1) and (2) present the results of the grouped regressions. In the group where the main suppliers are not SOEs, the digital infrastructure policies reduce the *SA* at the 1% level. This indicates that the effect of the digital infrastructure policies in alleviating financing constraints for Non-SOEs is more pronounced in firms that do not receive support from SOE suppliers. Similarly, when grouping by whether the main customers in the supply chain are SOEs, columns (3) and (4) show the results of the grouped regressions. In the group where the main customers are not SOEs, the digital infrastructure policies significantly reduce the *SA*. This suggests that the effect of the digital infrastructure policies in alleviating financing constraints for Non-SOEs is more significant in firms that do not receive support from SOE customers.

**Table 14:** Heterogeneity test of the impact differences of supply chain support.

	(1) The main suppliers include SOEs	(2) The main suppliers do not include SOEs	(3) The main customers include SOEs	(4) The main customers do not include SOEs
	*SA*	*SA*	*SA*	*SA*
*Policy*	−0.022	−0.003^***^	−0.016	−0.003^**^
	(−1.038)	(−2.677)	(−1.629)	(−2.548)
Controls	Yes	Yes	Yes	Yes
Constant	−5.035^***^ (−3.934)	−4.239^***^ (−17.069)	−7.551^***^ (−6.080)	−4.192^***^ (−16.929)
*Year & Industry & Area*	Yes	Yes	Yes	Yes
*R* ^2^ *_adj*	0.652	0.134	0.553	0.133
Observations	118	10611	187	10542

Table 14 presents the heterogeneity test of the impact differences of supply chain support. The superscripts ***, **, and * indicate statistical significance at 1%, 5% and 10% levels (two-tailed), respectively. We use a robust estimation and report t-values below the coefficients.

### 4.6. Further tests

#### 4.6.1. Do digital infrastructure policies attract Non-SOEs?

The impact of digital infrastructure policies on alleviating local fiscal burdens has improved the quality of fiscal revenue and the regional financial levels. This has, in turn, eased the financing constraints for Non-SOEs within the jurisdiction. Has the favorable impact of these policies attracted more Non-SOEs to relocate to areas where digital infrastructure policies are being implemented? *∆pl* represents whether the registration location of Non-SOEs has changed compared to the previous period. Columns (1) and (2) of Table 15 show the regression results of digital infrastructure policies and *∆pl*. The digital infrastructure policies (*Policy*) positively promote changes in the registration locations of Non-SOEs, and *∆Policy* is also significantly positively correlated with *∆pl*.

**Table 15:** Further tests.

	(1)	(2)	(3)
	∆*pl*	∆*pl*	VFI
*Policy*	0.197^*^		−0.005^***^
	(1.941)		(−26.388)
∆*Policy*		0.194^*^	
		(1.778)	
Controls	Yes	Yes	Yes
Constant	18.182	17.775^*^	1.868^***^
	(1.527)	(1.726)	(62.697)
*Year & Industry & Area*	Yes	Yes	Yes
*R* ^2^ *_adj*	0.223	0.228	0.952
Observations	7785	7566	10729

Table 15 presents the further test results. We specifically examined whether digital infrastructure policies positively influenced the changes in the registration locations of Non-SOEs and whether these policies helped alleviate local vertical imbalances. The superscripts ***, **, and * indicate statistical significance at 1%, 5% and 10% levels (two-tailed), respectively. We use a robust estimation and report t-values below the coefficients.

#### 4.6.2. Do digital infrastructure policies create vertical fiscal imbalances?

Hypothesis H1a suggests that digital infrastructure policies might exacerbate local vertical fiscal imbalances and reduce credit availability for private firms. However, the regression results do not support this hypothesis. Additionally, Column (3) of Table 15 presents the regression tests of the relationship between digital infrastructure policies and vertical fiscal imbalances. The findings indicate that digital infrastructure policies (*Policy*) are significantly and negatively associated with vertical fiscal imbalances (VFI) at the 1% level. This means that such policies help alleviate local vertical fiscal imbalances, thereby refuting Hypothesis H1a.

## 5. Conclusions

This study holds substantial economic significance and value. Economically, it reveals that digital infrastructure policies can optimize local fiscal structures by alleviating fiscal burdens, improving the quality of fiscal revenue, and enhancing the regional financial ecosystem. This not only contributes to the sustainable development of local finances but also provides new financing channels for Non-SOEs, which is of great help to the growth of the private economy. For instance, by reducing the financial constraints of Non-SOEs, these policies can promote the investment and innovation of enterprises, thereby stimulate economic vitality and promote industrial upgrading and transformation. Moreover, the study finds that digital infrastructure policies can attract the agglomeration of Non-SOEs without causing vertical fiscal imbalances. This is conducive to promoting the rational flow of resources among regions, narrowing regional economic development gaps, and achieving more balanced economic development. From a policy perspective, the research provides a scientific basis for governments at all levels to formulate and implement digital infrastructure policies. It helps governments better understand the impact of these policies on the economy and make more targeted policy adjustments to promote the healthy development of the digital economy and the private sector, which is of far – reaching significance for China to achieve high-quality economic development.

### 5.1. Policy recommendations

The findings provide actionable insights for policymakers to optimize digital infrastructure policies and alleviate financing constraints for Non-SOEs, supported by both empirical evidence and real-world case studies:

Provincial governments play a pivotal role in implementing digital infrastructure policies, as evidenced by Table 12, where policies issued by provincial authorities exhibit stronger effects on reducing financing constraints compared to lower-level agencies. For example, Zhejiang Province’s provincial-level “Digital Economy Innovation Hub” integrated fiscal subsidies, credit guarantee systems, and big data credit scoring to reduce financing costs for Non-SOEs (Zhejiang Provincial Government Report, 2023). Policymakers should replicate this model by establishing cross-departmental task forces to synchronize digital infrastructure deployment with fiscal reforms, such as expanding tax incentives for firms adopting digital technologies.
